# Appendicular neoplasms and consequences in patients undergoing surgery for suspected acute appendicitis

**DOI:** 10.1007/s00384-020-03673-0

**Published:** 2020-07-08

**Authors:** M. D. M. Bolmers, J. de Jonge, C. C. van Rossem, A. A. W. van Geloven, W. A. Bemelman, and on behalf of the Snapshot Appendicitis Collaborative Study group, G. J. van Acker, B. Akkermans, G. J. Akkersdijk, G. D. Algie, J. H. Allema, C. S. Andeweg, N. Appeldoorn, J. G. van Baal, C. M. den Bakker, S. A. Bartels, C. van den Berg, B. Boekestijn, F. C. den Boer, D. Boerma, A. L. van den Boom, M. C. Boute, S. A. Bouwense, J. Bransen, F. A. van Brussel, O. R. Busch, S. M. de Castro, H. A. Cense, C. Croese, T. van Dalen, I. Dawson, E. van Dessel, R. Dettmers, N. Dhar, F. Y. Dohmen, K. W. van Dongen, P. van Duijvendijk, R. R. Dulfer, B. J. Dwars, J. P. Eerenberg, M. van der Elst, E. van den Ende, L. M. Fassaert, J. T. Fikkers, J. W. Foppen, E. J. Furnee, F. P. Garssen, M. F. Gerhards, H. van Goor, J. S. de Graaf, L. J. Graat, J. Grootr, A. C. van der Ham, J. F. Hamming, J. T. Hamminga, E. van der Harst, J. Heemskerk, A. Heijne, J. T. Heikens, E. Heineman, R. Hertogs, E. van Heurn, L. C. van den Hil, A. G. Hooftwijk, C. C. Hulsker, D. R. Hunen, M. S. Ibelings, J. M. Klaase, R. Klicks, L. Knaapen, R. T. Kortekaas, F. Kruyt, S. Kwant, S. S. Lases, T. Lettinga, A. Loupatty, R. A. Matthijsen, R. C. Minnee, B. Mirck, L. Mitalas, D. Moes, A. M. Moorman, V. B. Nieuwenhuijs, G. A. Nieuwenhuizen, P. D. Nijk, J. M. Omloo, A. G. Ottenhof, H. W. Palamba, D. L. van der Peet, I. T. Pereboom, P. W. Plaisier, A. P. van der Ploeg, M. H. Raber, M. M. Reijen, H. Rijna, C. Rosman, R. M. Roumen, R. F. Scmitz, Velden P. A. van der Schouten, W. H. Scheurs, T. A. Sigterman, H. J. Smeets, D. J. Sonnevled, M. N. Sosef, S. F. Spoor, L. P. Stassen, L. van Steensel, E. Stortelder, J. Straatman, H. J. van Susante, D. E. Suykerbuyk de Hoog, C. Terwisscha van Scheltinga, B. R. Toorenvliet, P. C. Verbeek, M. Verseveld, J. H. Volders, M. R. Vriens, P. W. Vriens, B. C. Vrouenraets, B. J. van de Wall, J. A. Wegdam, E. Westerduin, J. J. Wever, N. A. Wijfels, B. P. Wijnhoven, T. A. Winkel, D. van der Zee, A. M. Zeillemaker, C. Zietse

**Affiliations:** 1 Department of surgery, Tergooi Hospital Hilversum, P.O. Box 10016, 1201 DA Hilversum, The Netherlands; 2 Department of Surgery, Maasstad Hospital, Rotterdam, The Netherlands; 3 Department of surgery, Academic Medical Center, Amsterdam, The Netherlands

**Keywords:** Appendix, Acute appendicitis, Neuroendocrine tumors (NET)

## Abstract

**Introduction:**

In patients treated with an appendectomy for acute appendicitis, the specimen is generally sent for histological evaluation. In an era of increasing non-operative treatment for acute appendicitis, it is important to know the incidence, the diagnostic accuracy, and treatment consequences of appendicular neoplasms that are found in acute appendicitis. We hypothesize that pre- and intra-operative parameters might predict an appendicular neoplasm.

**Methods:**

Data was used from our previous prospective observational cohort study. All patients undergoing surgery for suspected acute appendicitis were included. The primary outcome was the incidence of appendicular neoplasms in patients operated for acute appendicitis. Secondary outcomes were pre-operative diagnostics and imaging outcomes, intra-operative surgical judgment, and postoperative management and outcome. Possible predictors of an appendicular neoplasm were identified and used in multivariable logistic regression. Patients with an appendicular neoplasm were followed for 3 years after initial appendectomy.

**Results:**

A total of 1975 patients underwent surgery for suspected acute appendicitis and in 98.3% (1941/1975) the appendix was removed. In 1.5% (30/1941) of these patients, an appendicular neoplasm was found. Among the malignant neoplasms, the majority were grade 1 neuroendocrine tumors (NET) in 65% (13/20). On pre-operative imaging, there was no suspicion of malignancy. In three cases, there was an intra-operative suspicion of malignancy. Multivariable analysis showed only age as an independent predictor for appendicular neoplasms. No recurrent or new malignancy was found during follow-up.

**Discussion:**

The incidence of appendicular neoplasm in patients undergoing an acute appendectomy is very low and clinical risk factors could not be identified.

## Introduction

Emergency appendectomy for acute appendicitis is the most frequently performed acute surgical procedure in both children and adults. Obstruction of the appendicular lumen is the most frequent cause of inflammation of the appendix [1]. Usually, the obstruction is caused by either lymphoid hyperplasia or fecal impaction, but neoplasms may also be present. For this reason, it is common practice that all resected specimens are histologically evaluated, regardless of intra-operative judgment of the macroscopic appearance. An increasing number of studies evaluate the non-operative treatment of selected patients with simple acute appendicitis [2, 3], which implies that after successful antibiotic treatment, the appendix cannot be histologically evaluated and theoretically a neoplasm may remain undiagnosed in the patient. Primary neoplasms of the appendix are rare, less than 1% of patients are diagnosed with a neoplasm after appendectomy for acute appendicitis [4–6]. There is a wide spectrum of appendicular neoplasms, ranging from benign adenomas to primary adenocarcinomas of the appendix or mucinous cystic adenomas, which can evolve into peritoneal pseudomyxoma.

Current Dutch guideline states that all appendices should be send for histological evaluation following the acute appendectomy, independently of intra-operative surgical judgment. Because of the high incidence of acute appendicitis, the macro-economic costs of histological evaluation are significant. We hypothesize that it may only be necessary to send the specimen to the pathologist when a macroscopic abnormal appendix is found during surgery in selected patients.

The aim of this study is to evaluate the incidence of appendicular neoplasms discovered at histological evaluation in patients undergoing surgery for suspected acute appendicitis, and to identify possible pre- and intra-operative factors associated with the presence of an appendicular neoplasm. Secondly, we are interested in the different types of appendicular neoplasms and the corresponding postoperative management strategy. We hypothesize that pre-operative imaging and intra-operative macroscopic evaluation of the appendix are sufficient to detect clinically relevant appendicular neoplasm and that selective histological evaluation can be safely applied.

## Methods

A consecutive prospective observational snapshot audit was performed in 62 hospitals in the Netherlands. All hospitals that provided acute surgical care were invited to participate. This included academic, pediatric, and general community hospitals (teaching and non-teaching). The study was designed and led by surgical residents, who together with house officers collected the data. All consecutive patients undergoing surgery for suspected acute appendicitis were included in a period of 2 months (June and July 2014). Patients were treated according to the local protocol of the participating hospitals, based on the national guideline [6]. No adjustments on treatment were imposed for participating hospitals. This study was designed as an audit of appendicitis treatment and outcome in the country.

The study methods are explained in more detail elsewhere [7].

### Appendicular neoplasms

In this study, the presence of appendicular neoplasms discovered at histological evaluation was assessed in all adults and children (< 18 years of age) in whom the pre-operative clinical diagnosis was acute appendicitis. For further analysis, the total initial cohort that underwent surgery for suspected acute appendicitis was divided in two groups, to find possible pre- and intra-operative factors associated with the presence of an appendicular neoplasm. Group 1: patients with an appendicular neoplasm and group 2: patients without an appendicular neoplasm.

In the patients with an appendicular neoplasm, intra-operative judgment, types of appendicular neoplasms at histological evaluation, and the postoperative management were evaluated. These patients were followed for 3 years after initial appendectomy.

### Pre-operative imaging and intra-operative evaluation of the appendix

Pre-operative diagnostic imaging consisted of an ultrasound, computed tomography (CT) scan, or magnetic resonance imaging (MRI). Complicated appendicitis on pre-operative imaging was defined as an inflammatory mass with or without abscess or appendicitis with visible perforation.

During surgery, appendices were scored as uncomplicated or complicated appendicitis prior to resection. Uncomplicated appendicitis was defined as an inflamed but non-perforated appendix. Intra-operative complicated appendicitis was defined as gangrenous or perforated appendicitis with or without peritonitis. All resected specimens were sent for histological evaluation.

### Data extraction and outcome parameters

Pre-operative data, imaging results, intra-operative evaluation of the appendix, 30-day postoperative course, and histological results of each participating hospital were prospectively collected. The primary outcome was the incidence of appendicular neoplasms in patients that received an appendectomy for suspected acute appendicitis and had their appendix removed. Secondary outcomes were pre-operative imaging, intra-operative surgical judgment for complicated appendicitis and appendix suspicious for malignancy, and postoperative adjuvant treatment and diagnostics, in patients with an appendicular neoplasm.

### Statistical analysis

Normally distributed data was presented as mean with standard deviation (SD) and non-normally distributed data as median with interquartile range (IQR). Continuous variables were analyzed with the Mann-Whitney-Wilcoxon test and categorical data were analyzed using the Chi-square test or Fisher’s exact test. *P* < 0.05 was considered statistically significant. Possible pre- and intra-operative factors associated with the presence of an appendicular neoplasm were identified and used in multivariable logistic regression. All data was analyzed using IBM SPSS statistics, version 22.0 (IBM Corp., Armonk, NY, USA).

## Results

**Fig. 1:**
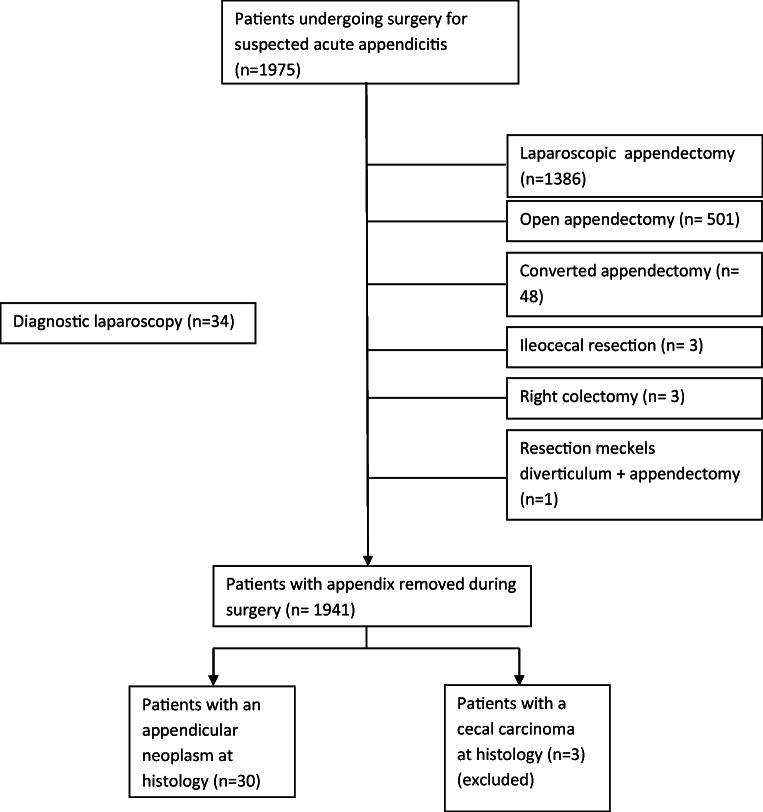
Flowchart of all patients in present analysis

A total of 1975 patients underwent surgery for suspected acute appendicitis in the 2-month study period. Of these patients, 98.3% (1941/1975) had their appendix removed (Fig. 1). In 1.5% (30/1941) of these patients, an appendicular neoplasm was discovered at histological evaluation and three patients were diagnosed with a cecal carcinoma. These three patients are excluded. The total cohort was divided in two groups for further analysis. Group 1: patients with an appendicular neoplasm (*n* = 30) and group 2: patients without an appendicular neoplasm (*n* = 1945). All demographics are shown in Table 1.

**Table 1:** Pre-operative data of patients with an appendicular neoplasm (*n* = 30) and patients without a neoplasms in initial cohort (*n* = 1945)

		(Group 1) Appendicular neoplasm (*n* = 30)	(Group 2) No appendicular neoplasm (*n* = 1945)	*p* value
Age, years (med, IQR)		50.5 (25.8;70)	29 (16;47)	< 0.001^a^
< 18 years (*n*, %)		2 (6.7)	539 (27.7)	0.01^b^
Sex, male (*n*, %)		16 (53.3)	1006 (51.7)	0.86^b^
ASA (*n*, %)	I–II	29 (96.7)	1891 (97.2)	0.57^c^
	III–IV	1 (3.3)	54 (2.8)	
Pre-hosp symptom time (*n*, %)	< 2 days	20 (66.7)	1499 (77.1)	0.1^b^
	> 2 days	10 (33.3)	399 (20.5)	
Pre-hosp symptom time (mean, SD)		2.7 (2.4)	1.96 (1.8)	0.096^d^
Migration of pain (*n*, %)		9 (30)	795 (40.9)	0.18^b^
Biochemics	White blood cell Count, 10˄9/L (mean, SD)	12.2 (4)	14.1 (4.8)	0.032^d^
	CRP, mg/L (med, IQR)	43.5 (13.5;87.3)	36 (62.8;75)	0.784^a^

^a^Mann-Whitney *U*

^b^Chi-square

^c^Fishers exact

^d^Levene’s test

### Pre-operative data and the incidence of appendicular neoplasms

The median age of the patients with an appendicular neoplasm was 50.5 years (IQR 25.8;70), compared with 29 years (IQR 16;47) in the patients without a neoplasm (*p* < 0.001). In the first group, two patients were < 18 years old, which was significantly less than 27.7% (539/1945) in the latter group (*p* = 0.01). No significant differences were found in sex, pre-hospital symptom time, and migration of pain (Table 1*)*.

### Pre-operative imaging and intra-operative evaluation of appendicular neoplasms

In all patients with an appendicular neoplasm at histological evaluation, pre-operative imaging was performed, consisting of an ultrasound in 66.6% (20/30), a CT scan alone in 6.6% (2/30), and ultrasound and CT scan in 23.3% (7/30) and in one patient an ultrasound followed by MRI was done. None of the 30 neoplasm were diagnosed with pre-operative imaging. Intra-operative examination identified in 3 out of the 30 neoplasm correctly of which one was an adenocarcinoma. Imaging showed uncomplicated appendicitis in 73.3% (22/30) of these patients without any suspicion for a neoplasm (Table 2*)*. In these 22 patients, adenoma, NET, and goblet cell carcinoid were found in the resected specimens. None of these patients had an adenocarcinoma. In five patients, pre-operative imaging showed complicated appendicitis without suspicion of a neoplasm. In these patients, all types of appendicular neoplasms were represented. Imaging results were inconclusive in 10% (3/30) of the patients and in their appendices an adenoma or NET was present. During intra-operative evaluation, uncomplicated appendicitis was reported in 60% (18/30) of the patients. Of these patients, 55.6% (10/18) had a NET, seven patients had an adenoma, and the remaining patient was diagnosed with a goblet cell carcinoid. In the remaining 40% (12/30), the intra-operative evaluation was complicated appendicitis. Pre-operative imaging and intra-operative diagnosis did not match completely, but both patients with an adenocarcinoma were pre- and intra-operatively were diagnosed with complicated appendicitis. In total, complicated appendicitis at pre-operative imaging or at intra-operative evaluation was not associated with the presence of a neoplasm (Table 2).

**Table 2:** Pre-operative imaging and intra-operative evaluation of patients with an appendicular neoplasm (*n* = 30) and patients without a neoplasm in initial cohort (*n* = 1945)

		(Group 1) Appendicular neoplasm (*n* = 30)	(Group 2) No appendicular neoplasm (*n* = 1945)	*p* value
Pre-operative diagnosis on imaging (*n*, %)	Uncomplicated appendicitis	22 (73.3)	1504 (77.3)	0.81*
	Complicated appendicitis	5 (16.7)	309 (15.9)	
Suspicion of malignancy (*n*, %)		0	NA	–
Intra-operative diagnosis (*n*, %)	Uncomplicated appendicitis	18 (60.0)	1314 (67.6)	0.43^*^
	Complicated appendicitis	12 (40.0)	631 (32.4)	
Suspicion of malignancy (*n*, %)		3	NA	–

*Fishers exact test

Multivariable regression analysis showed that increasing age was slightly associated with the presence of an appendicular neoplasm (OR 1.03; 1.01–1.05; *P* = 0.005) (Table 3).

**Table 3:** Multivariable analysis

	Total cohort (*n* = 1975)		
	OR	95% CI	*p* value
Age (per year)	1.03	1.01–1.05	0.005
> 18 years (versus > 18 years)	1.83	0.36–9.36	0.47
White blood cell count (10˄9/L)	0.93	0.85–1.01	0.08

### Types of appendicular neoplasms and additional management

Of all patients with an appendicular neoplasm, the neoplasm was a benign adenoma in 33.3% (10/30) and was malignant in 66.7% (20/30). These malignancies mainly consisted of a grade 1 (NET) in 65% (13/20), followed by a goblet cell carcinoid in 25% (5/20), an adenocarcinoma in 5% (1/20), and a mixed adeno-neuroendocrine carcinoma in 5% (1/20) (Table 4*)*. Table s 5 and 6 show the additional management of the appendicular neoplasms. This varied widely, 10/30 (33.3%) did not have either adjuvant imaging or therapy, and 9/30 (30%) had additional resection, consisting of two ileocecal resections and seven right-sided colectomies. These resections were planned after discussion in the institutional tumor board. No complications were reported in patients receiving additional treatment.

**Table 4:** Type of neoplasm

Type of neoplasm (*n* = 30)	*n* (%)
Adenoma	10 (33.3)
Grade 1 NET	13 (43.3)
Gobletcell carcinoid	5 (16.7)
Adenocarcinoma	1 (3.3)
Mixed adeno-neuroendocrine carcinoma	1 (3.3)

**Table 5:** Postoperative diagnostic imaging

Neoplasma	None	CT scan	Colonoscopy	Colonoscopy + CT	SPECT scan
Adenoma	3	1	4	2	0
Grade1 NET	6	1	1	0	1
Gobletcell carcinoid	1	0	0	0	0
Adenocarcinoma	0	0	1	0	0
Mixed adeno-neuroendocrine carcinoma	0	0	0	0	0
Total	10	2	6	2	1

**Table 6:** Postoperative adjuvant treatment

Neoplasma	Ileocecal resection	Right colectomy
Adenoma	0	0
Grade1 NET	2	2
Gobletcell carcinoid	0	4
Adenocarcinoma	0	0
Mixed adeno-neuroendocrine carcinoma	0	1
Total	2	7

### Follow-up/tumor recurrences

In one of nine patients that had additional resection, lymph node metastases were found of an adenocarcinoma, and adjuvant chemotherapy was administered. A total of 35 lymph nodes were collected during right colectomy of which 2 had metastasis. During initial work-up and surgery, this patient was diagnosed with complicated appendicitis. In this patient, no suspicion of malignancy was expressed on pre-operative CT scan or intra-operative evaluation of the appendix. The remaining eight resections did not yield extra tumor load. A total of 11 patients were exposed to an initial regimen of follow-up that ranged from a CT scan to a SPECT CT scan with colonoscopy, 6 months after initial appendectomy. Three years after initial appendectomy, no recurrent neoplasm was found in all 30 patients when we reviewed patients’ medical charts and all patients were alive.

## Discussion

In our large multicenter snapshot study, we showed that the overall incidence of appendicular neoplasms and malignancy, specifically in patients operated for acute appendicitis, is very low: 0.5% for benign neoplasms and 1.0% for malignancies. This is consistent with literature showing incidence rates of appendicular neoplasms between 0.54 and 1.7 [4, 8, 9].

We hypothesized, based on earlier studies [10, 11], that pre- or intra-operative parameters might contribute to neoplasm recognition. In this study however, we were not able to demonstrate this. Only increasing age was slightly indicative of an appendicular neoplasm.

Pre-operative imaging was not discriminative, most likely because neoplasms of the appendix are relatively small and can have a variable radiological appearance [12]. Suspicion of complicated appendicitis on pre-operative imaging showed no tendency towards a higher incidence of a neoplasm. The cecal carcinomas were not identified on pre-operative CT imaging. The intra-operative presence of complicated appendicitis showed no relation to appendicular neoplasms. In only three patients, the intra-operative suspicion of malignancy was expressed and confirmed in histological evaluation. However, the study was not designed for malignancy recognition by the operating surgeon, and surgeons were not trained to assess the appendix for a neoplasm systematically.

A large variety of neoplasms can occur in the appendix, both benign and malignant. Murphy et al. published a useful classification system [5]. In our study, we used a simplified classification system, dealing with the most common malignancies. We divided neoplasms in grade 1 NET with a very good 5-year survival rate between 88 and 100% [13], goblet cell carcinoid with a 5-year survival rate between 18 and 86% [14], and (epithelial) adenocarcinomas with survival rates between 11 and 42% [14], as this may have clinical consequences for their respective adjuvant treatment.

In most malignant neoplasms, small low-grade neuroendocrine tumors at the tip of the appendix are diagnosed, generally with no clinical consequences [15]. However, patients suffering from larger and more poorly differentiated tumors may benefit from a right hemicolectomy as adjuvant treatment. The purpose of this colonic resection can be both therapeutic and prognostic, although remaining tumor or lymph node metastasis is seldom found.

Because of the rarity and lack of knowledge, there is variety in management among physicians and it is difficult to predict the value of additional surgery for the individual patient. In some cases, patients might receive unnecessary additional treatment and are exposed to the risks of a second surgery. Nowadays there are guidelines for (adjuvant) treatment of neuroendocrine tumors (NETs), but there is less consensus on more rare subtypes like goblet cell carcinoid [16].

In patients undergoing elective appendectomy after non surgically managed appendicular inflammatory mass or in patients with a pathologic appearing appendix on imaging, the incidence of neoplasms is much higher than in acute appendectomy [17, 18].

We showed in our study that a large variety of adjuvant treatment strategies were used for the various neoplasms, all patients were discussed in an institutional tumor board. The different treatment strategies may have been caused by different tumor boards and patient preferences. Only one patient (0.05%) had persistent malignant disease with nodal involvement in the additional right colectomy specimen, for which he was treated with adjuvant chemotherapy.

Eight out of nine patients had an additional resection without extra tumor yield, consisting of the grade 1 NET and goblet cell carcinoid. Majority of these patients received complementary pre-operative imaging. Inevitably, all these patients were exposed to the morbidity and mortality risks of colonic resections. After 3 years of follow-up, no recurrent disease was found, and all patients were alive.

In this study population, the clinical consequences of pathologic examination of the appendix specimen seems low. However, eight patients had additional resection without any remaining tumor found in the specimen. These patients were put at risk for surgery without having the benefits of the additional resection.

We have not been able to show either pre- or intra-operative risk factors for appendicular neoplasms apart from increasing age. However, our study was not designed to prove specific risk factors and further research on this subject is imminent as more and more patients are non-operatively managed for suspicion of uncomplicated appendicitis. We suggest that operating surgeons should be trained in potential neoplasm recognition and after future studies one might refrain from histological evaluation in patients with uncomplicated appendicitis.

## References

[1] Burkitt DP (1971). The aetiology of appendicitis. Br J Surg.

[2] Paajanen H, Gronroos JM, Rautio T, Nordstrom P, Aarnio M, Rantanen T, Hurme S, Dean K, Jartti A, Mecklin JP (2013). A prospective randomized controlled multicenter trial comparing antibiotic therapy with appendectomy in the treatment of uncomplicated acute appendicitis (APPAC trial). BMC Surg.

[3] Podda M, Cillara N, Di Saverio S, Lai A, Feroci F, Luridiana G, Agresta F, Vettoretto N (2017). Antibiotics-first strategy for uncomplicated acute appendicitis in adults is associated with increased rates of peritonitis at surgery. A systematic review with meta-analysis of randomized controlled trials comparing appendectomy and non-operative management with antibiotics. Surgeon : J Royal Coll Surg Edinburgh Ireland.

[4] Connor SJ, Hanna GB, Frizelle FA (1998). Appendiceal tumors: retrospective clinicopathologic analysis of appendiceal tumors from 7,970 appendectomies. Dis Colon Rectum.

[5] Murphy EM, Farquharson SM, Moran BJ (2006). Management of an unexpected appendiceal neoplasm. Br J Surg.

[6] Bakker OJ, Go PM, Puylaert JB, Kazemier G, Heij HA (2010). Guideline on diagnosis and treatment of acute appendicitis: imaging prior to appendectomy is recommended. Ned Tijdschr Geneeskd.

[7] van Rossem CC, Bolmers MD, Schreinemacher MH, van Geloven AA, Bemelman WA, Snapshot Appendicitis Collaborative Study G (2016). Prospective nationwide outcome audit of surgery for suspected acute appendicitis. Br J Surg.

[8] Machado NO, Chopra P, Pande G (2004). Appendiceal tumour--retrospective clinicopathological analysis. Trop Gastroenterol : Off J Digest Dis Foundation.

[9] Bucher P, Mathe Z, Demirag A, Morel P (2004). Appendix tumors in the era of laparoscopic appendectomy. Surg Endosc.

[10] Loftus TJ, Raymond SL, Sarosi GA, Croft CA, Smith RS, Efron PA, Moore FA, Brakenridge SC, Mohr AM, Jordan JR (2017). Predicting appendiceal tumors among patients with appendicitis. J Trauma Acute Care Surgery.

[11] Lietzen E, Gronroos JM, Mecklin JP, Leppaniemi A, Nordstrom P, Rautio T, Rantanen T, Sand J, Paajanen H, Kaljonen A (2019). Appendiceal neoplasm risk associated with complicated acute appendicitis-a population based study. Int J Color Dis.

[12] Ganeshan D, Bhosale P, Yang T, Kundra V (2013). Imaging features of carcinoid tumors of the gastrointestinal tract. AJR Am J Roentgenol.

[13] Madani A, van der Bilt JD, Consten EC, Vriens MR, Borel Rinkes IH (2015). Perforation in appendiceal well-differentiated carcinoid and goblet cell tumors: impact on prognosis? A systematic review. Ann Surg Oncol.

[14] McGory ML, Maggard MA, Kang H, O’Connell JB, Ko CY (2005). Malignancies of the appendix: beyond case series reports. Dis Colon Rectum.

[15] O’Donnell ME, Badger SA, Beattie GC, Carson J, Garstin WI (2007). Malignant neoplasms of the appendix. Int J Color Dis.

[16] Pape UF, Niederle B, Costa F, Gross D, Kelestimur F, Kianmanesh R, Knigge U, Oberg K, Pavel M, Perren A (2016). ENETS consensus guidelines for neuroendocrine neoplasms of the appendix (excluding goblet cell carcinomas). Neuroendocrinology.

[17] Wright GP, Mater ME, Carroll JT, Choy JS, Chung MH (2015). Is there truly an oncologic indication for interval appendectomy?. Am J Surg.

[18] Mallinen J, Rautio T, Gronroos J, Rantanen T, Nordstrom P, Savolainen H, Ohtonen P, Hurme S, Salminen P (2019). Risk of Appendiceal neoplasm in periappendicular abscess in patients treated with interval appendectomy vs follow-up with magnetic resonance imaging: 1-year outcomes of the peri-appendicitis acuta randomized clinical trial. JAMA Surg.

